# Survival of esophageal and gastric cancer patients with adjuvant and palliative chemotherapy—a retrospective analysis of a register-based patient cohort

**DOI:** 10.1007/s00228-020-02883-3

**Published:** 2020-05-05

**Authors:** Isabella Ekheden, Fereshte Ebrahim, Halla Ólafsdóttir, Pauline Raaschou, Björn Wettermark, Roger Henriksson, Weimin Ye

**Affiliations:** 1 Department of Medical Epidemiology and Biostatistics, Karolinska Institutet, Box 281, SE171 77 Stockholm, Sweden; 2 Regional Cancer Centre Stockholm Gotland, Stockholm County Council, Västgötagatan 2, 102 39 Stockholm, Sweden; 3 Cancer Theme, Karolinska University Hospital, 171 64 Stockholm, Sweden; 4 Department of Medicine Solna, Clinical Epidemiology Section, Karolinska Institutet, 171 76 Stockholm, Sweden; 5 Department of Laboratory Medicine (LABMED), H5, Division of Clinical Pharmacology, C1:68, Karolinska University Hospital, Huddinge, 141 86 Stockholm, Sweden; 6 Department of Pharmacy, Uppsala University, Box 580, 751 23 Uppsala, Sweden; 7 Department of Radiation Sciences, Umeå University, 901 87 Umeå, Sweden

**Keywords:** Esophageal cancer, Gastric cancer, Chemotherapy, Adjuvant, Palliative, Survival

## Abstract

**Purpose:**

The survival of esophageal and gastric cancer patients treated with chemotherapy is rarely assessed outside of clinical trials. Therefore, we compared the effectiveness of various curative or palliative chemotherapy regimens on the survival of esophageal and gastric cancer patients in a “real world” clinical setting.

**Methods:**

We identified a cohort of 966 incident esophageal and gastric cancer patients in Stockholm/Gotland County (a low-risk Western population) during 2008–2013. Patients who received chemotherapy with curative intention (*n* = 279) and palliative intention (*n* = 182) were analyzed separately. Using Cox proportional hazards regression models, we estimated hazard ratios (HRs) with 95% confidence intervals (CIs) and adjusted for the potential confounding factors: age, sex, TNM stage, radiotherapy, comorbidity, marital status, education, income, and country of birth.

**Results:**

In esophageal cancer patients with curative treatment intention, we observed a higher hazard for death among patients who received carboplatin-fluorouracil compared to patients who received cisplatin-fluorouracil, corresponding to a HR of 2.18 (95% CI 1.09–4.37). Conversely, in patients with cancer in the gastroesophageal junction who had a curative treatment intention at diagnosis, we observed a reduced hazard for death among those who received fluorouracil-oxaliplatin, compared to patients who received cisplatin-fluorouracil (HR 0.28; 95% CI 0.08–0.96).

**Conclusion:**

Among patients with esophageal cancer who received treatment with curative intention, cisplatin-fluorouracil was associated with better survival compared to carboplatin-fluorouracil, while patients with gastroesophageal junction cancer who were treated with cisplatin-fluorouracil had worse survival compared to fluorouracil-oxaliplatin.

**Electronic supplementary material:**

The online version of this article (10.1007/s00228-020-02883-3) contains supplementary material, which is available to authorized users.

## Introduction

Patients with esophageal and gastric cancer (EC and GC) have a very poor prognosis with an overall mortality to incidence ratio of 0.89 and 0.76, respectively, according to an IARC (International Agency for Research on Cancer) report [1]. The poor prognosis is mainly due to delayed diagnosis caused by late presentation of symptoms, when the disease usually has reached an advanced, metastatic stage. At this stage, the gold standard treatment, curative surgery, is no longer beneficial for the majority of patients [2]. In fact, only about 20–30% of EC [3] and GC [4] patients are eligible for curative surgery at diagnosis. In addition to radical tumor resection, neoadjuvant chemoradiotherapy or chemotherapy for EC patients and perioperative chemotherapy or adjuvant chemotherapy for GC patients have been established in clinical practice as an add-on treatment alternative to prolong survival [5], except for a minority of patients with cervical EC who can be cured with chemoradiotherapy alone [6]. Despite advances in surgical techniques and addition of chemotherapy over the past decades, the survival of EC and GC patients has not improved substantially and the mortality remains high [7–9]. Many potential new chemotherapies are currently explored in clinical trials, continuously including fit and willing patients [10–14]. However, the majority of patients are excluded from these clinical trials due to their advanced disease, poor physical conditions, and/or co-morbidities [15]. The use and outcome of chemotherapy in these patients can differ both regarding efficacy and safety compared to pre-registration reports from study patients [16]. Furthermore, chemotherapy can be used in other combinations and with other co-medications than previously studied, which might influence the effectiveness and/or safety in these patients. Follow-up data on post-marketing chemotherapy effectiveness and patient survival in the clinical, “real-world” setting are limited. Such information is of interest for regulators, caregivers, and patients. Unfortunately, previous post-marketing studies on EC and GC from the “real-world” setting are few and their results are inconclusive [17–21].

Therefore, we compared the effect of chemotherapy on the survival of esophageal and gastric cancer patients in a clinical setting with “real-world” data from retrospective registers in Stockholm and Gotland County during 2008–2016.

## Material and methods

### Data collection—population, time periods, and variables

The Stockholm and Gotland region in Sweden was comprised of almost 2.4 million people at the end of 2018 according to the census from Statistics Sweden. Current national guidelines for treatment of patients with esophageal or gastric cancer have been established collaboratively through Regional Cancer Centers (Regionala cancercentrum i samverkan) [22].

A cohort of patients diagnosed with esophageal or gastric cancer between 1 January 2008 and 31 December 2013 was constructed (Supplementary Fig. 1) from the Research Database at Regional Cancer Centre in Stockholm/Gotland, described in detail elsewhere [23–25]. The cohort was followed until death (all-cause mortality), emigration, or end of follow-up (31 December 2016), whichever occurred first. Individual-level data on exposure, outcome, and adjustment variables were obtained using a unique identifier, the National Registration Number, for linkages with the data collected in six national and three regional registers between 1 January 2001 and 31 December 2016 [26] (Supplementary Fig. 2).

We applied the tenth Swedish edition of the International Classification of Diseases (ICD-10), the second edition of the International Classification of Diseases in Oncology (ICD-O) for topography of the tumor, the Swedish version of the Systematized Nomenclature of Medicine II (SNOMED II) for tumor morphology, and the Anatomical Therapeutic Chemical (ATC) classification for exposure to drugs (Supplementary Table 1). We calculated the Charlson Comorbidity Index Score according to previously updated weights [27].

We included patients with curative or palliative treatment intention at diagnosis from the quality register “Nationellt kvalitetsregister för matstrups- och magsäckscancer” (NREV), and excluded patients with no tumor-specific treatment or missing treatment intention from further analyses. We analyzed the curative and palliative treatment groups separately. We divided the cancer patients into three groups depending on cancer site: the esophagus, gastroesophageal junction, or stomach. We excluded patients with tumor stage T0/Tis and missing T stage or Tx. Only chemotherapy treatment initiated within 6 months from diagnosis until 3 weeks after the start of the treatment was included in the analysis.

### Statistical analysis

Comparisons of patient characteristics between groups were made using Wilcoxon two-sample test for continuous variables, Chi-squared test for categorical variables with ≥ 5, and Fisher’s exact test for categorical variables with less than five observations.

We used Kaplan-Meier graphs to illustrate survival curves and log-rank test to compare the difference of survival curves. In addition, Cox proportional hazards regression was employed to estimate hazard ratio (HR) with 95% confidence intervals (CI) to compare the effect of chemotherapy on survival separately for patients treated with curative and palliative intention. We tested the proportional hazards assumption and used stratification when this assumption was not met. We adjusted for potential confounding factors such as age (continuous), sex (men/women), and tumor stage (T1 + 2/T3 + 4, missing/N−/N+, missing/M−/M+) in a minimally adjusted model and added radiotherapy (unknown or missing/yes), comorbidity (0/1–5), marital status (missing/married/unmarried or divorced or widowed), education (missing/high and medium level/low level), income (missing/below median/equal to or above median), and country of birth (Sweden/other) in the fully adjusted model. We assessed the influence of unknown tumor stage by a sensitivity analysis including patients with unknown tumor stage.

We utilized SAS 9.4 (SAS Institute Inc., Cary, NC, USA) for data management and analyses, and R Studio 1.0.153 (RStudio, Inc.) for producing Kaplan-Meier survival curves.

## Results

In the final cohort of 966 patients, the mean age at cancer diagnosis was 66.7 years among patients in the curative treatment group and 69.9 among patients in the palliative treatment group (Table 1). More men than women were diagnosed with gastroesophageal cancer both in the curative and in the palliative treatment groups (Table 1).

**Table 1:** Characteristics of the study subjects in a register-based cohort study on treatment in esophageal and gastric cancer patients in Stockholm county, Sweden 2008–2016 (*n* = 966)

Variables	Palliative treatment (*n* = 453) *N* (%)	Curative treatment (*n* = 513) *N* (%)	*p* value
Mean age (S.D.), years at diagnosis	69.9 (12.7)	66.7 (11.0)	< 0.0001*
Sex			0.2040**
Male	297 (65.6)	356 (69.4)	
Female	156 (34.4)	157 (30.6)	
Tumor site			<0.0001**
Esophagus	229 (50.6)	184 (35.9)	
Gastroesophageal junction	66 (14.6)	94 (18.3)	
Stomach	158 (34.9)	235 (45.8)	
Cancer subtype			< 0.0001**
Esophageal squamous cell carcinoma	110 (24.3)	84 (16.4)	
Esophageal adenocarcinoma	110 (24.3)	98 (19.1)	
Adenocarcinoma in gastroesophageal junction	62 (13.7)	90 (17.5)	
Gastric adenocarcinoma	155 (34.2)	226 (44.1)	
Missing	16 (3.5)	15 (2.9)	
T stage			< 0.0001**
T1	11 (2.4)	57 (11.1)	
T2	30 (6.6)	97 (18.9)	
T3	220 (48.6)	282 (55.0)	
T4	192 (42.4)	77 (15.0)	
N stage			< 0.0001**
N negative	81 (17.9)	192 (37.4)	
N positive	313 (69.1)	315 (61.4)	
Unknown/not assessed/missing (Nx or missing)	59 (13.0)	6 (1.2)	
M stage			< 0.0001**
M negative	166 (36.6)	464 (90.5)	
M positive	270 (59.6)	44 (8.6)	
Unknown/not assessed/missing (Tx or Nx)	17 (3.8)	5 (1.0)	
Occupation			< 0.0001**
White collar	40 (8.8)	64 (12.5)	
Blue collar	53 (11.7)	134 (26.1)	
Pink collar^d^	79 (17.4)	165 (32.2)	
Age ≥ 65 years	211 (46.6)	98 (19.1)	
Missing	70 (15.5)	52 (10.1)	
Survival time^a^, days. Median (IQR)	167 (255)	776 (1383)	< 0.0001*
Palliative/curative radio-chemotherapy			0.0412**
Yes	176 (37.6)	167 (32.6)	
No or missing	277 (61.1)	346 (67.4)	
Status at end of follow-up, 31 December 2016			< 0.0001*
Alive	15 (3.3)	151 (29.4)	
Died 2015–2016, missing cause of death	9 (2.0)	39 (7.6)	
Died from other causes	42 (9.3)	38 (7.4)	
Died from esophageal and junction cancer	213 (47.0)	138 (26.9)	
Died from gastric cancer	174 (38.4)	147 (28.7)	
Follow-up time^b^, days. Median (IQR)	210 (298)	654 (1194)	< 0.0001*
Education			0.244**
Low (primary school)	143 (31.6)	147 (28.7)	
Middle (upper secondary school)	164 (36.2)	219 (42.7)	
High (university)	110 (24.3)	134 (26.1)	
Missing	36 (8.0)	13 (2.5)	
Smoking			0.8541**
Never	109 (24.1)	124 (24.2)	
Ex-smoker (quit > 1 year ago)	75 (16.6)	95 (18.5)	
Current (and quit < 1 year ago)	98 (21.6)	113 (22.0)	
Unknown or missing	171 (37.8)	181 (35.3)	
Marital status			0.2520**
Married	228 (50.3)	273 (53.2)	
Not married	82 (18.1)	69 (13.5)	
Divorced	84 (18.5)	103 (20.1)	
Widowed	57 (12.6)	68 (13.3)	
Missing	2 (0.4)	0 (0.0)	
Annual income percentiles^c^			0.3701**
0–24th percentile	98 (21.6)	88 (17.2)	
25–49th percentile	102 (22.5)	121 (23.6)	
50–74th percentile	114 (25.2)	139 (27.1)	
75–100th percentile	138 (30.5)	164 (32.0)	
Missing	1 (0.2)	1 (0.2)	
Place of birth			0.2897**
Sweden	351 (77.5)	375 (73.1)	
Europe	67 (14.8)	91 (17.7)	
Africa/Asia/Oceania/America	35 (7.7)	47 (9.2)	
Charlson Comorbidity Index Score			0.0816**
0	294 (64.9)	371 (72.3)	
1	35 (7.7)	32 (6.2)	
2	72 (15.9)	69 (13.5)	
≥ 3	52 (11.5)	41 (8.0)	

^a^Survival time defined as time in years from diagnosis to death date

^b^Defined as time in years from start of chemotherapy until death, emigration or end of follow-up (31st of December 2016), whichever occurred first

^c^Annual income in 1000 SEK, percentile groups: 0-24th percentile: [0–1126), 25-49th percentile [1126–1440), 50-74th percentile [1440–2016), 75-100th percentile [2016–28,636]

^d^Service sector jobs

*Wilcoxon Two-sample test, T approximation, Two-sided Pr > |Z|

**Chi-squared test

In the curative treatment group, patients had less advanced tumor stage at diagnosis, a higher proportion with a current occupation (due to younger age), and a longer survival after diagnosis (Table 1). Fewer patients in the curative group received radiotherapy in addition to chemotherapy compared to the palliative group (Table 1).

At the end of follow-up, 31 December 2016, about 30% of the patients in the curative-intention group were alive, while only 3% of the palliative-intention group had survived (Table 1).

The distribution of other demographic variables (Table 1) and use of anti-inflammatory drugs and drugs against peptic ulcer/gastro-esophageal reflux disease (GERD) up to 1 year before diagnosis (Supplementary Table 2) were not statistically different between the curative and palliative treatment groups.

The distribution of TNM stages among patients with different chemotherapy regimens in the curative-treatment group was similar, except for patients with cancer in the esophagus treated with cisplatin-fluorouracil who had more advanced T and N stages at diagnosis. Mean age at diagnosis and percentage of patients who received radiotherapy were different between groups except radiotherapy for patients with stomach cancer, where very few received radiotherapy. There was a higher proportion of male than female patients with cancer in the gastroesophageal junction in the cisplatin-fluorouracil or epirubicin-oxaliplatin-capecitabine group than other chemotherapy groups, while the distribution of males was similar between chemotherapy groups in patients with cancer in the esophagus and stomach (Table 2).

**Table 2:** Characteristics for patients with cancer in the esophagus, gastroesophageal junction, and stomach who received chemotherapy with curative intention within 6 months from diagnosis (*n* = 279)

Cancer site and chemotherapy groups	Cohort	Stage			Mean age (SD)	%Radiotherapy	%Male
		(T1 + T2)/(T3 + T4)	%Npos	%Mpos			
Esophagus, *N*	132						
Cisplatin-fluorouracil	85	14/71	81	6	63.3 (7.5)	78	74
Fluorouracil-oxaliplatin	23	4/19	61	4	67.6 (6.0)	65	78
Carboplatin-fluorouracil	14	3/11	64	7	67.9 (6.7)	93	79
Other chemotherapy	10	3/7	50	0	71.4 (6.4)	70	70
*p* value		*p* = 0.01	*p* < 0.01	*p* = 1.00	*p* < 0.01	*p* < 0.01	*p* = 0.98
Gastroesophageal junction, *N*	59						
Cisplatin-fluorouracil	34	6/28	76	12	60.4 (9.4)	82	91
Fluorouracil-oxaliplatin	13	2/11	54	0	64.5 (9.0)	54	69
Epirubicin-oxaliplatin-capecitabine	7	2/5	86	14	69.1 (4.7)	14	100
Other chemotherapy	5	3/2	20	0	70.2 (11.3)	40	40
*p* value		*p* = 0.09	*p* = 0.12	*p* = 0.75	*p* = 0.05	*p* < 0.01	*p* = 0.03
Stomach, *N*	88						
Epirubicin-oxaliplatin-capecitabine	71	24/47	54	7	58.7 (10.4)	3	61
Fluorouracil-irinotecan	8	0/8	88	0	66.9 (7.2)	0	88
Other chemotherapy	9	4/5	56	33	70.3 (9.0)	0	56
*p* value		*p* = 0.17	*p* = 0.18	*p* = 0.20	*p* < 0.01	*p* = 0.40	*p* = 0.48

**Fig. 1:**
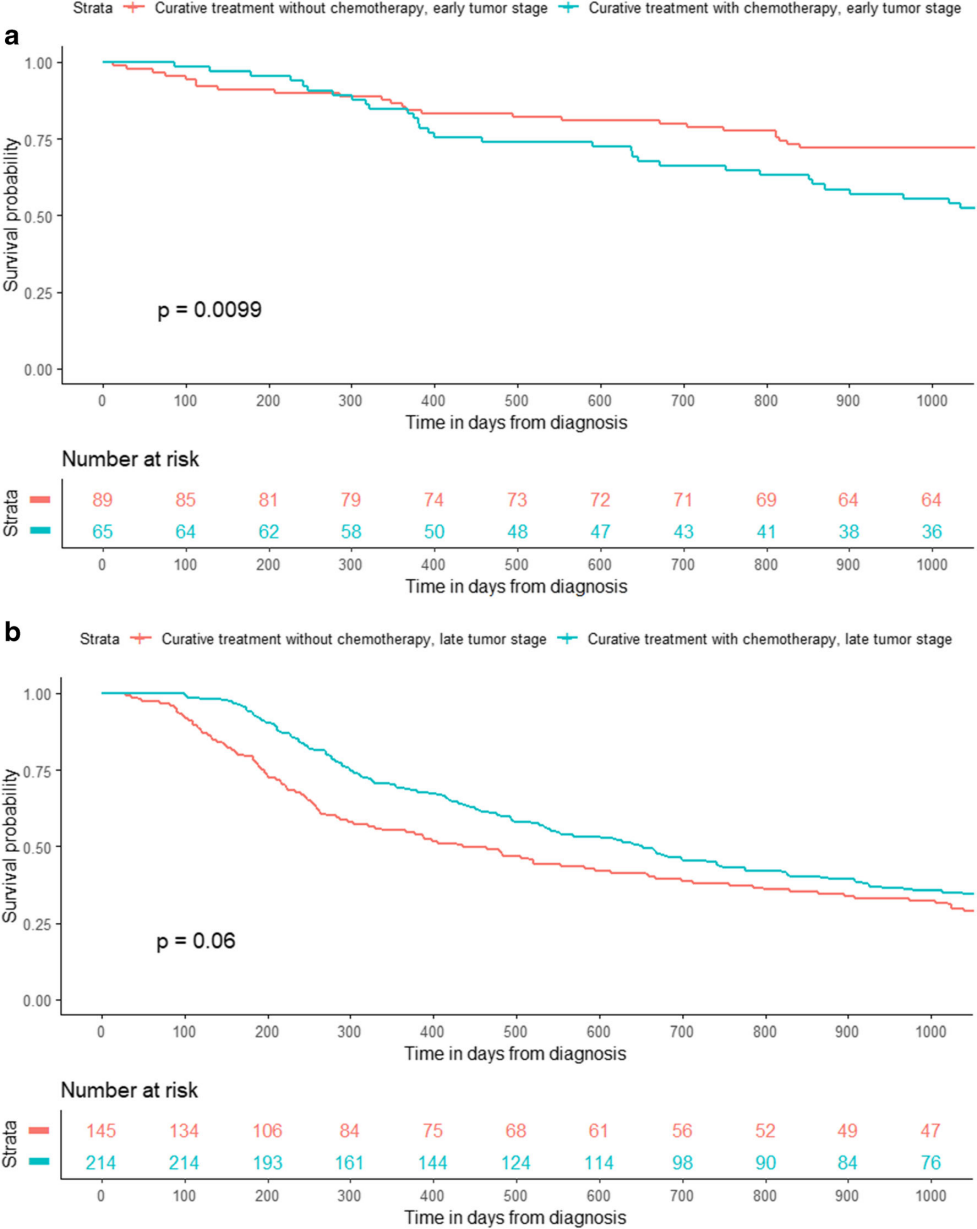

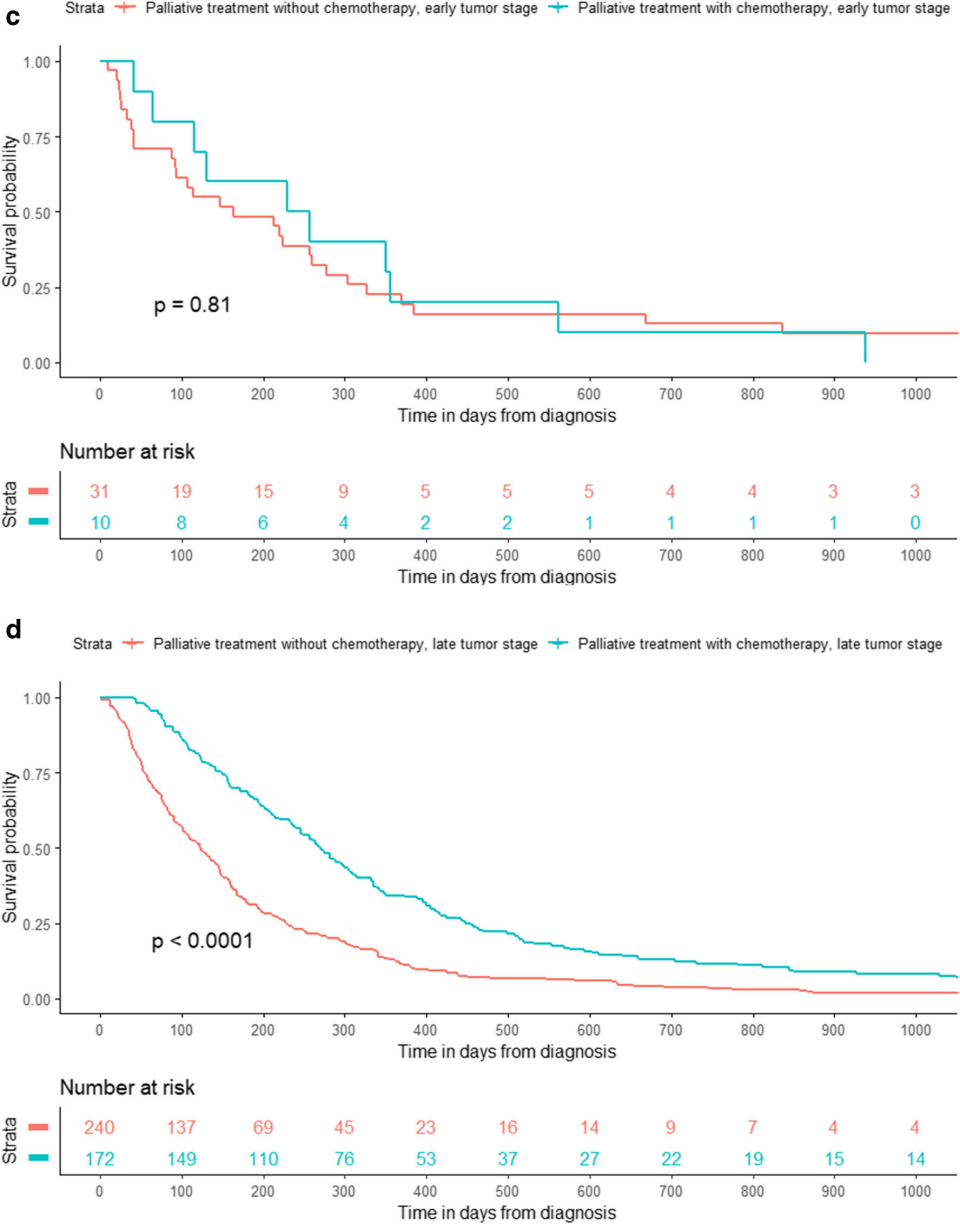
Kaplan-Meier graphs of survival in days after chemotherapy in esophageal and gastric cancer patients. Patients treated with curative intention who received chemotherapy within the first 6 months from diagnosis compared to those who did not receive chemotherapy, for those with early tumor stage *n* = 154 (**A**), and with late tumor stage *n* = 359 (**B**), separately, and corresponding graphs for patients with palliative treatment, for those with early tumor stage *n* = 41 (**C**) and late tumor stage *n* = 412 (**D**), separately

In early-stage patients with curative treatment intention at diagnosis, initial survival was not significantly different, however, long-term survival seemed to be better in patients without chemotherapy vs. with chemotherapy (*p* = 0.0099) (Fig. 1A). The difference in survival between those who received chemotherapy vs. those without chemotherapy was not statistically significant neither in the curative-intention group with late tumor stage (Fig. 1B) nor in the palliative-intention group with early tumor stage (Fig. 1C). However, patients with late-stage tumor who received chemotherapy in the palliative-intention group had a clearly better survival (*p* < 0.0001) than patients without chemotherapy (Fig. 1D).

**Fig. 2:**
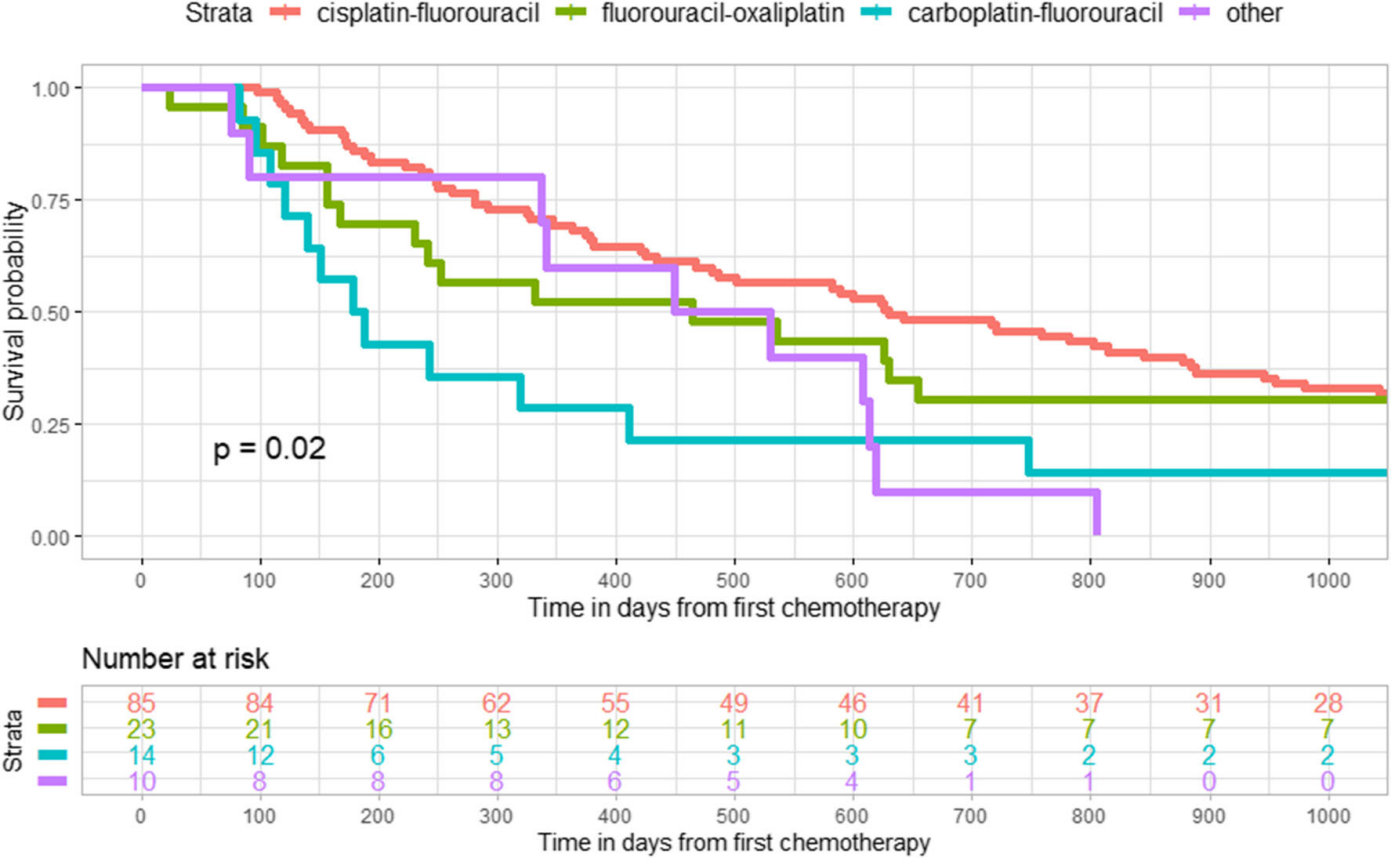
Kaplan-Meier graph of survival in days after chemotherapy among patients with cancer in the esophagus who received treatment with curative intention (*n* = 132)

**Fig. 3:**
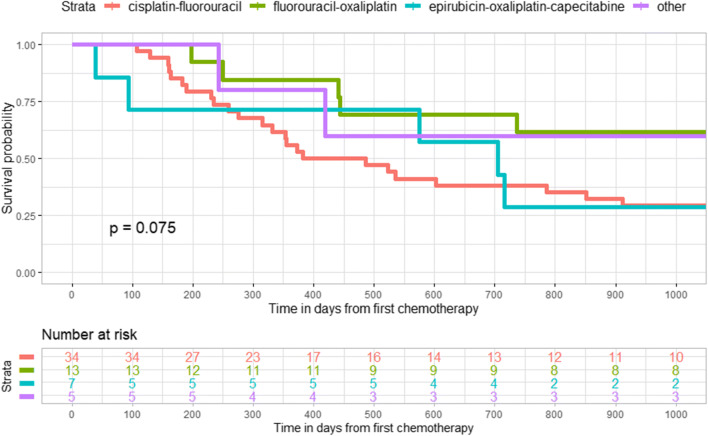
Kaplan-Meier graph of survival in days after chemotherapy among patients with cancer in the gastroesophageal junction who received treatment with curative intention (*n* = 59)

**Fig. 4:**
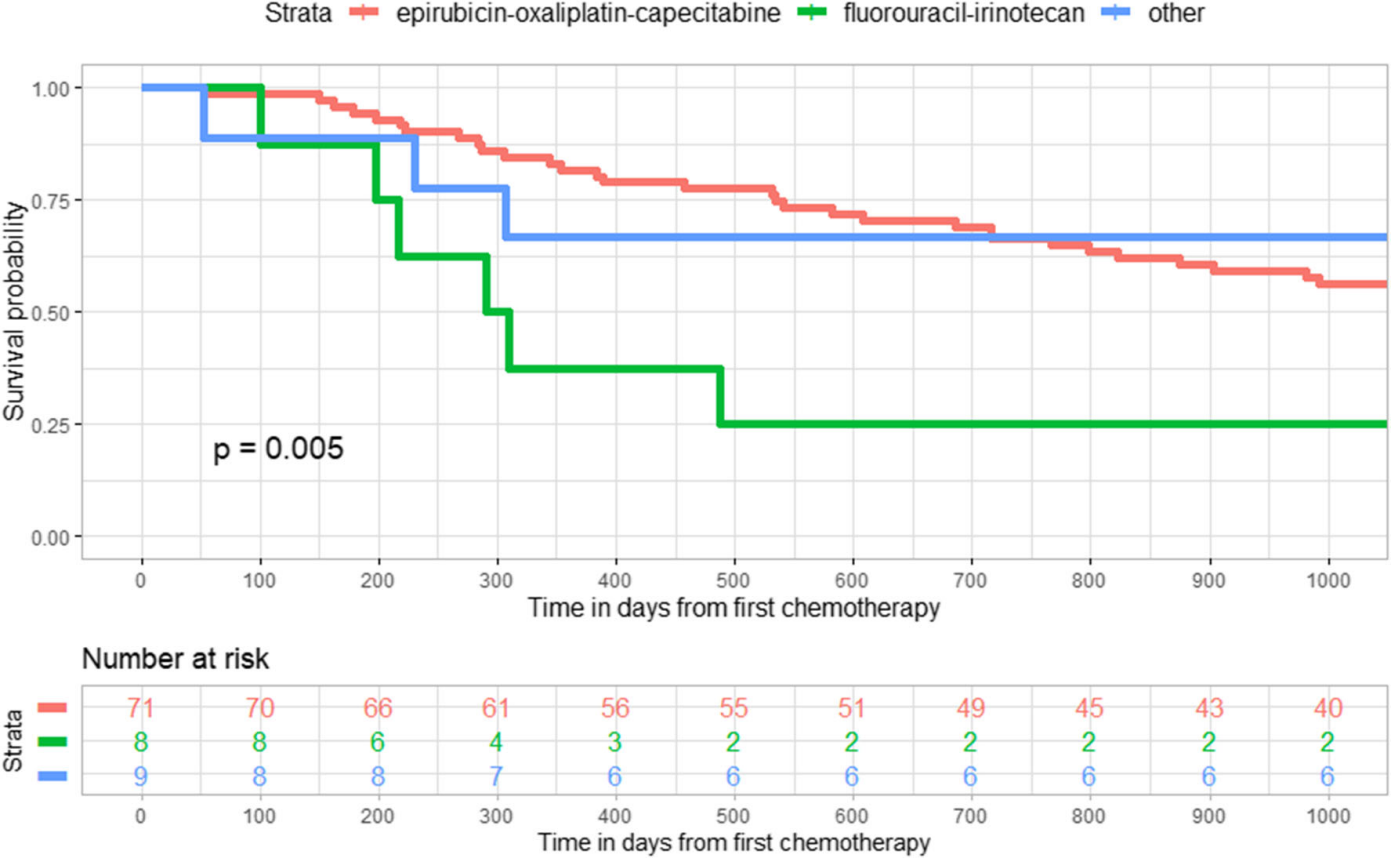
Kaplan-Meier graph of survival in days after chemotherapy among patients with cancer in the stomach who received treatment with curative intention (*n* = 88)

We observed a statistically significant survival benefit by choice of chemotherapy (cisplatin-fluorouracil) in the unadjusted Kaplan-Meier graph among esophageal cancer patients in the curative-intention group (Fig. 2), while no such difference existed for patients with cancer in the gastroesophageal junction **(**Fig. 3**)**. In addition, there was a statistically significant difference in survival among patients with gastric cancer in the curative-intention group by choice of chemotherapy (epirubicin-oxaliplatin-capecitabine or fluorouracil-irinotecan) (Fig. 4).

In the fully adjusted Cox regression model for the curative group, we could demonstrate a higher HR for death of 2.18 (95% CI 1.09–4.37) for patients with cancer in the esophagus who received carboplatin-fluorouracil compared to the reference group (cisplatin-fluorouracil). Similarly, a more than doubled HR for death, 2.23 (95% CI 1.02–4.91), was detected for those patients treated with other, more unusual chemotherapy (Table 3). Among those patients with cancer in the gastroesophageal junction who were treated with fluorouracil-oxaliplatin, we observed a lower HR of 0.28 (0.08–0.96) compared to cisplatin-fluorouracil. Among gastric cancer patients in the curative treatment group, none of the chemotherapy regimens were associated with better or worse survival in the fully adjusted Cox model (Table 3). These associations could not be confirmed in the palliative-intention group (Supplementary Table 3) due to a different choice of treatment than in the curative-intention group. In the palliative-intention group with cancer in the gastroesophageal junction, the fully adjusted Cox regression model showed a trend of higher HR for treatment with other, more unusual chemotherapy, HR 32.53 (95% CI 3.97–266.89) (Supplementary Table 3).

**Table 3:** Cohort size and hazard ratios for chemotherapy with curative intention within 6 months from diagnosis with cancer in the esophagus, gastroesophageal junction, or stomach (*n* = 279)

Chemotherapy groups by cancer site	Cohort *N*	Adjusted HR^a^	*p* value	Adjusted HR^b^	*p* value
Esophagus, *N*	132				
Cisplatin-fluorouracil	85	Ref.	Ref.	Ref	Ref.
Fluorouracil-oxaliplatin	23	1.53 (0.90–2.60)	0.12	1.28 (0.70–2.35)	0.43
Carboplatin-fluorouracil	14	2.33 (1.24–4.38)	0.01	2.18 (1.09–4.37)	0.03
Other chemotherapy	10	2.77 (1.34–5.73)	0.01	2.23 (1.02–4.91)	0.05
Gastroesophageal junction, *N*	59				
Cisplatin-fluorouracil	34	Ref.	Ref.	Ref.	Ref.
Fluorouracil-oxaliplatin	13	0.45 (0.16–1.25)	0.12	0.28 (0.08–0.96)	0.04
Epirubicin-oxaliplatin-capecitabine	7	0.76 (0.27–2.11)	0.60	0.34 (0.07–1.73)	0.20
Other chemotherapy	5	1.00 (0.25–4.06)	1.00	0.72 (0.15–3.46)	0.68
Stomach, *N*	88				
Epirubicin-oxaliplatin-capecitabine	71	Ref.	Ref.	Ref.	Ref.
Fluorouracil-irinotecan	8	2.64 (1.13–6.18)	0.03	2.26 (0.92–5.53)	0.07
Other chemotherapy	9	0.45 (0.15–1.36)	0.16	0.45 (0.14–1.40)	0.17

^a^Adjusted for age (continuous), sex, and TNM stage

^b^Additionally adjusted for radiotherapy, comorbidity, marital status, education, income, and country of birth

For comparison of chemotherapy choices, we performed separate sensitivity analyses in the curative-intention group (Supplementary Table 4) and palliative-intention group (Supplementary Table 5) including patients with unknown and missing tumor stage. The direction of the estimates did not change except for reduced HR for esophageal cancer patients treated with carboplatin-fluorouracil in the palliative-intention group, which was not statistically significant, and gastric cancer patients in the palliative-intention group treated with other, more unusual chemotherapy where the association was only marginally statistically significant.

## Discussion

Our results show that patients with curative treatment intention were younger than those with palliative treatment intention. This could reflect a better performance status, which is an essential part of being able to benefit from and tolerate multimodal curative treatment. The fact that these patients also received less radiotherapy than the palliative treatment group may have also contributed to shorten the time to curative surgery. We could confirm the large difference in survival between curative and palliative treatment patients, which is probably due to the effect of multimodal treatment including tumor resection, but the impact of tumor burden and metastatic pattern could not be excluded. We did not find a statistically different distribution of demographic variables between the curative and palliative treatment groups which is an indication of health care equity.

We found that chemotherapy (vs no chemotherapy) influenced survival for patients with early-stage tumors in the curative treatment group and late-stage tumors in the palliative treatment group, but not late cancer stage patients in the curative treatment group and early cancer stage patients in the palliative treatment group. A reason for an advantage in long-term survival among patients with early-stage tumors in the curative treatment group was likely due to a shorter median time to surgery among those without chemotherapy vs. with chemotherapy. On the contrary, patients in the late stage palliative treatment group did not have curative surgery and therefore the effect of chemotherapy is clearer. Similarly, the influence of curative surgery seems to attenuate the effect of chemotherapy in the late-cancer-stage patients with curative treatment. Very few patients with early cancer stage had a palliative treatment intention at diagnosis, probably since they were not eligible for curative treatment due to various reasons such as patient characteristics (frailty, age, and comorbidities) or tumor characteristics (bulky tumor, lymph node metastases, cellular/molecular markers). These characteristics also seem to attenuate the effect of chemotherapy.

In our unadjusted Kaplan-Meier graph, esophageal cancer patients in the curative treatment group had a higher survival rate if treated with cisplatin-fluorouracil and gastric cancer patients had a better prognosis if treated with epirubicin-oxaliplatin-capecitabine and fluorouracil-irinotecan compared with other chemotherapy regimens. There was no statistically significant difference in the Kaplan-Meier graph between the various curative chemotherapy regimens among patients with gastroesophageal junction cancer. A similar trend, although not statistically significant, was observed in the palliative treatment group. We are only aware of two previous clinical trials that have made a head-to-head comparison between some of the chemotherapy regimens in our study. The first is a randomized trial among esophageal cancer patients not eligible for surgery and they did not find a statistically significant increase of progression-free survival for patients who received cisplatin-fluorouracil compared to FOLFOX (oxaliplatin-fluorouracil-leucovorin) [28], which was in line with our results in the palliative-treatment group, but is contrary to our findings in the curative treatment group. Furthermore, the OE05 trial [29] did not find an increased survival in esophageal adenocarcinoma patients treated with neoadjuvant ECX (epirubicin-cisplatin-capecitabine) compared to cisplatin-fluorouracil, which is in line with our results for epirubicin-oxaliplatin-capecitabine in the gastroesophageal junction cancer patients. Unfortunately, it is unlikely that any future clinical trial will make a head-to-head comparison of the chemotherapy regimens in our study since they are outdated in the Western world. The combination of cisplatin-fluorouracil for esophageal cancer has been practiced in Sweden since the 1980s when proven effective in head and neck squamous cell carcinoma patients [30], and is the most common regimen seen in our study period, followed by the combination EOX (epirubicin-oxaliplatin-capecitabine), which is an equivalent [31] of the ECF/ECX regimen (epirubicin-cisplatin-fluorouracil/capecitabine) which was reported in 2006 in the MAGIC trial [13] to significantly improve disease-free and overall survival in gastroesophageal cancer patients compared to surgery alone. Since then, the clinical treatment practice has been changed to use preoperative chemoradiotherapy treatment according to the CROSS trial (carboplatin-paclitaxel) which was published in 2012 [11] or the neoadjuvant FLOT regimen (fluorouracil-leucovorin-oxaliplatin-docetaxel) which showed a superior survival rate to ECF/ECX in a publication in 2016 [12]. In the meanwhile, fluoropyrimidine/platinum (fluorouracil-cisplatin) based perioperative regimens are recommended for patients with gastroesophageal junction or gastric cancer according to the FFCD trial [5, 14]. Our study period precedes the large clinical trials such as CROSS and FLOT that have shaped current treatment guidelines. We therefore found a larger variation in treatment than one can probably find in more recent data. The older chemotherapy regimens that we have compared to each other in this study are most likely used in a much lesser extent today than during the study period but may still be in clinical use and gives important insight into the impact on survival of various chemotherapy regimens. Esophageal and gastric cancer patients with curative treatment who were eligible to receive the most common chemotherapy regimens (cisplatin-fluorouracil in esophageal cancer and epirubicin-oxaliplatin-capecitabine or fluorouracil-irinotecan in gastric cancer patients) had a higher survival rate than those patients who received other chemotherapies. The explanation may be that these patients were more fit with regard to patient and tumor characteristics so that they were perceived by the treating physician as being able to benefit from the standard treatment and to be able to tolerate it, which seems to be a very good prognostic marker. Patients who received more uncommon chemotherapy regimens had lower survival possibility, but since they were not eligible for the most common chemotherapy, maybe their prognosis should rather be compared with palliative treatment patients.

Our main finding is that, in our fully adjusted Cox model, patients with esophageal cancer who were treated with cisplatin-fluorouracil in the curative treatment group experienced a better survival compared to patients treated with carboplatin-fluorouracil, while patients with cancer in the gastroesophageal junction who received cisplatin-fluorouracil had worse survival than patients treated with fluorouracil-oxaliplatin. Since carboplatin has a more favorable toxicity profile than cisplatin, we interpret the result where carboplatin-fluorouracil–treated patients had a lower survival rate than those treated with cisplatin-fluorouracil as an effect of their underlying diminished capacity to tolerate the cisplatin-based regimen. It is possible that gastroesophageal junction cancer patients might tolerate the fluorouracil-oxaliplatin regimen better than cisplatin-fluorouracil, and so the better delivery of the chemotherapy regimen might result in the higher survival rate among these patients.

The implication of these findings is that the choice of chemotherapy may predict survival in patients with tumors in the esophagus and gastroesophageal junction that are treated with curative intention. Yet, we have no previous studies to compare these associations with and therefore would need to interpret these results cautiously until they have been validated in future studies.

### Strengths and limitations

Confounding by indication could explain the observed results although we have tried to decrease this risk by several methods, such as restricting the analysis to patients with known tumor stage. To further reduce the risk of confounding by indication, we looked at the curative-intention and palliative-intention groups separately. Thirdly, the study cohort is only from the Stockholm/Gotland area with reasonably similar treatment routines although small differences between hospitals may remain (80% of the patients were however treated at the three largest hospitals “Karolinska University Hospital Huddinge”, “Danderyd Hospital” and “Södersjukhuset”). Lastly, we classified the chemotherapy groups according to use of chemotherapy until 6 months after diagnosis. Analyzing chemotherapy used a longer time after diagnosis would have introduced a greater risk of confounding by indication since clinicians would have even more clinical information available to guide their chemotherapy regimen choice, an information that we could not access through registers.

The risk of differential misclassification of the main exposure and outcome is low since we collected data from registers that have high coverage and accuracy. Misclassification of the treatment intention at diagnosis is possible but we believe the risk for this will be low. Missing data on some of the exposures such as smoking, radiotherapy, and occupation as well as tumor stage was however an issue that we could not overcome. Due to missing data, we chose not to include smoking and occupation in the fully adjusted Cox regression model. This may lead to differential misclassification and either under- or overestimation of the association. Stratification by radiotherapy did not change hazard ratios significantly in the curative treatment group. We could unfortunately not analyze radiotherapy any further since we had no information about doses and regimens. We could not stratify the patients with the same chemotherapy according to dose and duration due to the large underlying variability. The large variability in dose and duration of chemotherapy is to try to reach similar exposures despite inter-individual differences in metabolism, elimination, tolerance, and effect. We tried to limit the effect of this by only including the first cycle of chemotherapy.

This study expands the current field in several aspects. To the best of our knowledge, this is the first study to make a head-to-head comparison of various “real-life” chemotherapy regimens on the survival of gastroesophageal cancer patients. It is not likely that any future clinical trial will make head-to-head comparisons of these chemotherapy regimens since they are not part of currently established guideline recommendations. However, they may still be in clinical use and comparing their effect on survival is of interest for both regulators, caregivers, and patients. Unique and high-quality information about which parenteral chemotherapy has been used in esophageal and gastric cancer patients in Stockholm/Gotland is one of the advantages of this study; this information is unfortunately not available in the nationwide Swedish registers. Another strength is the study base of clinical “real-world” patients, which includes patients commonly excluded from clinical trials. The time period 2008–2013 was of special interest as we believe there was a larger variation of chemotherapy than currently. Lastly, we could gather information about important prognostic factors such as tumor stage, co-morbidities, and socioeconomic status.

We conclude that esophageal cancer patients who received carboplatin-fluorouracil had a twofold higher HR for death compared to patients treated with cisplatin-fluorouracil in Stockholm/Gotland 2008–2013. Moreover, patients with cancer in the gastroesophageal junction treated with fluorouracil-oxaliplatin had a reduced HR for death compared to patients treated with cisplatin-fluorouracil.

## Electronic supplementary material

Supplementary Table 1 (DOCX 17 kb).

Supplementary Table 2 (DOCX 16 kb).

Supplementary Table 3 (DOCX 23 kb).

Supplementary Table 4 (DOCX 22 kb).

Supplementary Table 5 (DOCX 23 kb).

Supplementary figure 1 Flow-chart of patient selection of esophageal and gastric cancer patients in Stockholm/Gotland 2001-2013. (PNG 3467 kb).

High resolution image (TIF 247 kb).

Supplementary figure 2 Chart of registries used. Time period used for the construction of the cohort marked with green shade (2008-2016). (PNG 4201 kb).

High resolution image (TIF 375 kb).

## Data Availability

The data that support the findings of this study are available from Regional Cancer Center Stockholm/Gotland but restrictions apply to the availability of these data, which were used under license for the current study, and so are not publicly available. Data are however available from the authors upon reasonable request and with permission of Regional Cancer Center Stockholm/Gotland.
