# Supplementary material for: Survival of esophageal and gastric cancer patients with adjuvant and palliative chemotherapy—a retrospective analysis of a register-based patient cohort
Source: Eur J Clin Pharmacol. 2020 May 5;76(7):1029–41. doi: 10.1007/s00228-020-02883-3 (PMC7306049; doi:10.1007/s00228-020-02883-3)
Supplement: Supplementary file 1 — (DOCX 17 kb). [file 228_2020_2883_MOESM1_ESM.docx]

| Supplementary table 1 Codes used for cohort selection and/or statistical analyses. | | |
| --- | --- | --- |
| Coding system | **Variable explanation and start of variable registration** | **Codes used** |
| ICD7 | Localization of the tumor according to ICD7, 1958- | Cancer in the esophagus: 150X  Cancer in the gastroesophageal junction: 1511  Stomach cancer: 151X (not 1511) |
| ICD-O/2-10 | Localization of the tumor according to ICD-O/2 (with certain elements of ICD-10-SE), 1993- | Cancer in the esophagus: C15.X  Cancer in the gastroesophageal junction: C16.0  Stomach cancer: C16.1-C16.9 |
| ICD-10-SE | Main- and bidiagnoses in inpatient- or outpatient care, 2001- | https://www.socialstyrelsen.se/Lists/Artikelkatalog/Attachments/20021/2015-12-39.pdf |
| ULORSAK | The underlying illness that leads to the chain of events that ultimately lead to death, 1997- (ICD10) | Esophageal and junction cancer: C15.X-C16.0,  Gastric cancer: C16.1-C16.9 |
| SNOMEDO10 | Morphology of the tumor according to ICD-O/2, 1993- | Squamous cell carcinoma: 80702, 80703  Adenocarcinoma: 81402, 81403 |
| ATC | ATC-code according to WHO, 2005-07-01- | A02B (Drugs for peptic ulcer and gastro-esophageal reflux disease (GERD))  L01 (Antineoplastic agents),  L02 (Endocrine therapy),  M01A (Antiinflammatory and antirheumatic products, non-steroids),  N02BA01, B01AC06 (Acetylsalicylic acid) |
