# Supplementary material for: Survival of esophageal and gastric cancer patients with adjuvant and palliative chemotherapy—a retrospective analysis of a register-based patient cohort
Source: Eur J Clin Pharmacol. 2020 May 5;76(7):1029–41. doi: 10.1007/s00228-020-02883-3 (PMC7306049; doi:10.1007/s00228-020-02883-3)
Supplement: Supplementary file 2 — (DOCX 16 kb). [file 228_2020_2883_MOESM2_ESM.docx]

| **Supplementary table 2** Characteristics of drug use in study subjects in a register-based cohort study on treatment in esophageal and gastric cancer patients in Stockholm county, Sweden 2008-2016 (n=966). | | | |
| --- | --- | --- | --- |
| **Variables** | **Palliative treatment (n=453)**  **N (%)** | **Curative treatment (n=513)**  **N (%)** | ***P*-value** |
| Antiinflammatory drugs NSAID or ASA |  |  | 0.2973* |
| Missing | 207 (45.7) | 210 (40.9) |  |
| More than six months | 100 (22.1) | 110 (21.4) |  |
| Less than six months | 146 (32.2) | 193 (37.6) |  |
| Drugs for peptic ulcer and GERD |  |  | 0.1348* |
| Missing | 323 (71.3) | 367 (71.5) |  |
| More than six months | 66 (14.6) | 61 (11.9) |  |
| Less than six months | 64 (14.1) | 85 (16.6) |  |
| Chemotherapy regimens^a^, N | 182 | 279 | <0.0001** |
| Cisplatin-fluorouracil | 19 | 119 |  |
| Fluorouracil-oxaliplatin | 52 | 36 |  |
| Carboplatin-fluorouracil | 7 | 14 |  |
| Epirubicin-oxaliplatin-capecitabine | 7 | 78 |  |
| Fluorouracil-irinotecan | 62 | 8 |  |
| Fluorouracil | 15 | 0 |  |
| Other chemotherapy^b^ | 20 | 24 |  |
| ^a^ Within six months from diagnosis date.  ^b^ Single therapy with cisplatin, carboplatin, oxaliplatin, leuprorelin, cyclofosfamid, imatinib, anastrozole, methotrexate, gemcitabine, sorafenib, bicalutamide, hydroxycarbamide or combination therapy with paclitaxel+carboplatin, paclitaxel-carboplatin-anastrozole, epirubicin-oxaliplatin, etoposide-carboplatin, cisplatin-cetuximab, goserelin-bicalutamide.  *Chi-Square Test, **Fisher’s Exact Test, Two-sided Pr <= P | | | |
