# Supplementary material for: Survival of esophageal and gastric cancer patients with adjuvant and palliative chemotherapy—a retrospective analysis of a register-based patient cohort
Source: Eur J Clin Pharmacol. 2020 May 5;76(7):1029–41. doi: 10.1007/s00228-020-02883-3 (PMC7306049; doi:10.1007/s00228-020-02883-3)
Supplement: Supplementary file 3 — (DOCX 23 kb). [file 228_2020_2883_MOESM3_ESM.docx]

| **Supplementary table 3** Cohort size and hazard ratios for chemotherapy with palliative intention within six months from diagnosis with cancer in the esophagus, gastroesophageal junction or stomach (n=182). | | | | | | | |
| --- | --- | --- | --- | --- | --- | --- | --- |
| **Chemotherapy groups by cancer site** | **Cohort N** | **Adjusted HR^a^** | | | **P-value** | **Adjusted HR^b^** | **P-value** |
| **Esophagus, *p*-value** | 68 |  | | |  |  |  |
| Fluorouracil-oxaliplatin | 31 | Ref. | | | Ref. | Ref | Ref. |
| Cisplatin-fluorouracil | 19 | 0.67 (0.35-1.27) | | | 0.22 | 0.76 (0.37-1.55) | 0.45 |
| Carboplatin-fluorouracil | 7 | 1.04 (0.41-2.67) | | | 0.94 | 1.19 (0.38-3.72) | 0.76 |
| Other chemotherapy | 11 | 1.10 (0.47-2.61) | | | 0.82 | 1.59 (0.57-4.48) | 0.38 |
|  |  |  | | |  |  |  |
| **Gastroesophageal junction,  *p*-value** | 32 |  | | |  |  |  |
| Fluoruracil-oxaliplatin | 14 | Ref. | | | Ref. | Ref. | Ref. |
| Fluorouracil-irinotecan | 12 | 1.45 (0.57-3.67) | | | 0.44 | 1.03 (0.33-3.22) | 0.96 |
| Other chemotherapy | 6 | 14.38 (2.49-83.21) | | | <0.01 | 32.53 (3.97-266.89) | <0.01 |
|  |  |  | | |  |  |  |
| **Stomach,  *p*-value** | 82 |  | | |  |  |  |
| Fluorouracil-irinotecan | 50 | Ref. | | | Ref. | Ref. | Ref. |
| Fluorouracil | 15 | 1.32 (0.63-2.76) | | | 0.46 | 1.30 (0.59-2.86) | 0.52 |
| Fluorouracil-oxaliplatin | 7 | 1.69 (0.57-5.05) | | | 0.35 | 2.02 (0.58-7.03) | 0.27 |
| Epirubicin-oxaliplatin-capecitabine | 7 | 0.37 (0.14-0.99) | | | 0.05 | 0.44 (0.15-1.23) | 0.11 |
| Other chemotherapy | 3 | 3.06 (0.80-11.76) | | | 0.10 | 3.25 (0.71-14.82) | 0.13 |
|  |  | |  |  |  |  |  |
| ^a^ Adjusted for age (continuous), sex and TNM stage  ^b^ Additionally adjusted for radiotherapy, comorbidity, marital status, education, income and country of birth. | | | | | | | |
