# Supplementary material for: Survival of esophageal and gastric cancer patients with adjuvant and palliative chemotherapy—a retrospective analysis of a register-based patient cohort
Source: Eur J Clin Pharmacol. 2020 May 5;76(7):1029–41. doi: 10.1007/s00228-020-02883-3 (PMC7306049; doi:10.1007/s00228-020-02883-3)
Supplement: Supplementary file 4 — (DOCX 22 kb). [file 228_2020_2883_MOESM4_ESM.docx]

| **Supplementary table 4** Sensitivity analysis including missing/unknown tumor stage. Cohort size and hazard ratios for first chemotherapy within six months from diagnosis with cancer in the esophagus, gastroesophageal junction or stomach with curative treatment intention at diagnosis (n=286). | | | | | | | |
| --- | --- | --- | --- | --- | --- | --- | --- |
| **Chemotherapy groups by cancer site** | **Cohort N** | **Adjusted HR^a^** | | | **P-value** | **Adjusted HR^b^** | **P-value** |
| **Esophagus, *p*-value** | 134 |  | | |  |  |  |
| Cisplatin-fluorouracil | 87 | Ref. | | | Ref. | Ref | Ref. |
| Fluorouracil-oxaliplatin | 23 | 1.53 (0.90-2.61) | | | 0.12 | 1.27 (0.69-2.34) | 0.44 |
| Carboplatin-fluorouracil | 14 | 2.34 (1.24-4.40) | | | 0.01 | 2.21 (1.10-4.44) | 0.03 |
| Other chemotherapy | 10 | 2.75 (1.33-5.71) | | | 0.01 | 2.18 (0.99-4.80) | 0.05 |
|  |  |  | | |  |  |  |
| **Gastroesophageal junction,  *p*-value** | 60 |  | | |  |  |  |
| Cisplatin-fluorouracil | 35 | Ref. | | | Ref. | Ref. | Ref. |
| Fluorouracil-oxaliplatin | 13 | 0.45 (0.16-1.25) | | | 0.12 | 0.28 (0.08-0.96) | 0.04 |
| Epirubicin-oxaliplatin-capecitabine | 7 | 0.76 (0.27-2.11) | | | 0.60 | 0.34 (0.07-1.73) | 0.20 |
| Other chemotherapy | 5 | 1.00 (0.25-4.06) | | | 1.00 | 0.72 (0.15-3.46) | 0.68 |
|  |  |  | | |  |  |  |
| **Stomach,  *p*-value** | 92 |  | | |  |  |  |
| Epirubicine-oxaliplatin-capecitabine | 74 | Ref. | | | Ref. | Ref. | Ref. |
| Fluorouracil-irinotecan | 8 | 2.64 (1.13-6.18) | | | 0.03 | 2.26 (0.92-5.53) | 0.07 |
| Other chemotherapy | 10 | 0.45 (0.15-1.36) | | | 0.16 | 0.45 (0.14-1.40) | 0.17 |
|  |  | |  |  |  |  |  |
| ^a^ Adjusted for age (continuous), sex and tumour stage  ^b^ Additionally adjusted for radiotherapy, comorbidity, marital status, education, income and country of birth. | | | | | | | |
