# Supplementary figures and images for: Survival of esophageal and gastric cancer patients with adjuvant and palliative chemotherapy—a retrospective analysis of a register-based patient cohort
Source: Eur J Clin Pharmacol. 2020 May 5;76(7):1029–41. doi: 10.1007/s00228-020-02883-3 (PMC7306049; doi:10.1007/s00228-020-02883-3)

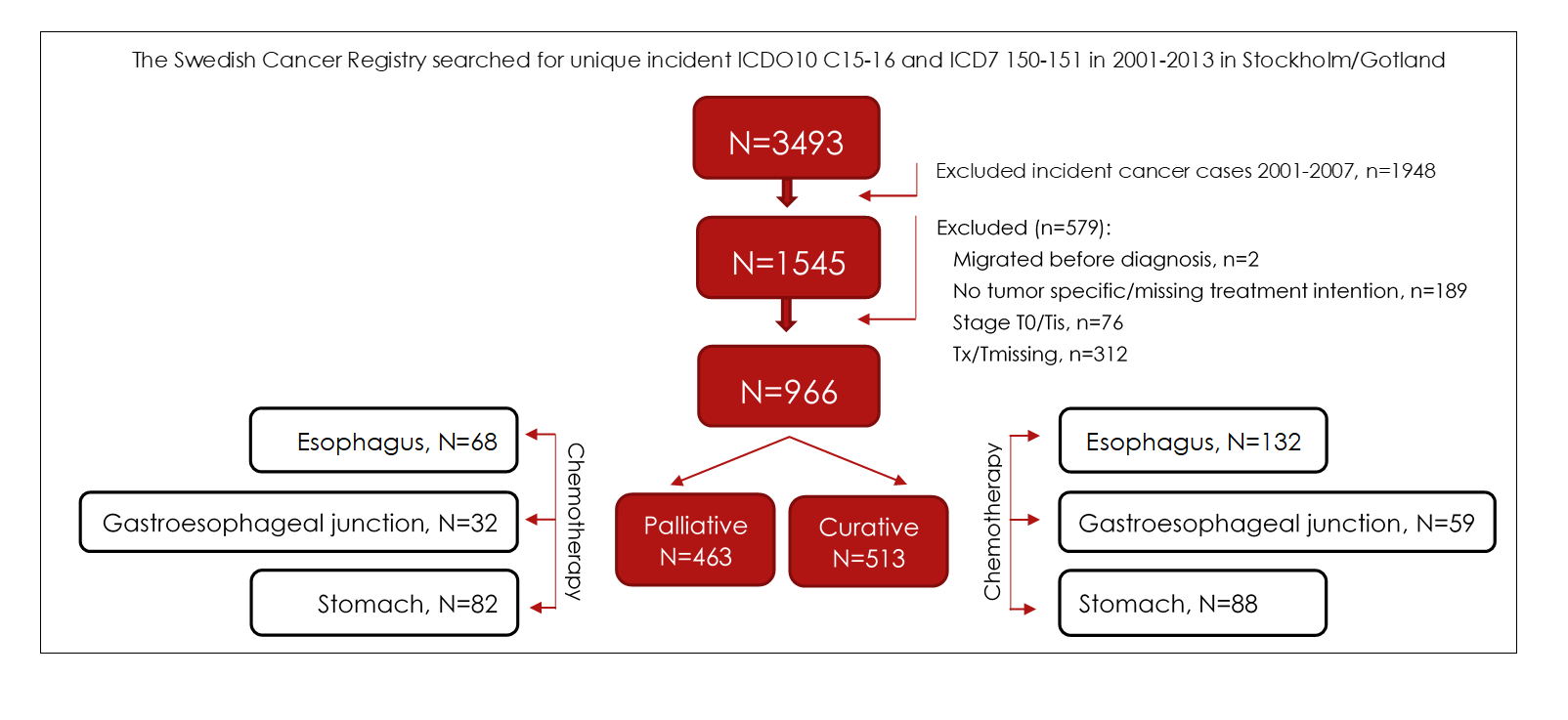

Supplement: Supplementary file 6 — Flow-chart of patient selection of esophageal and gastric cancer patients in Stockholm/Gotland 2001-2013. (PNG 3467 kb). [file 228_2020_2883_Fig5_ESM.png]

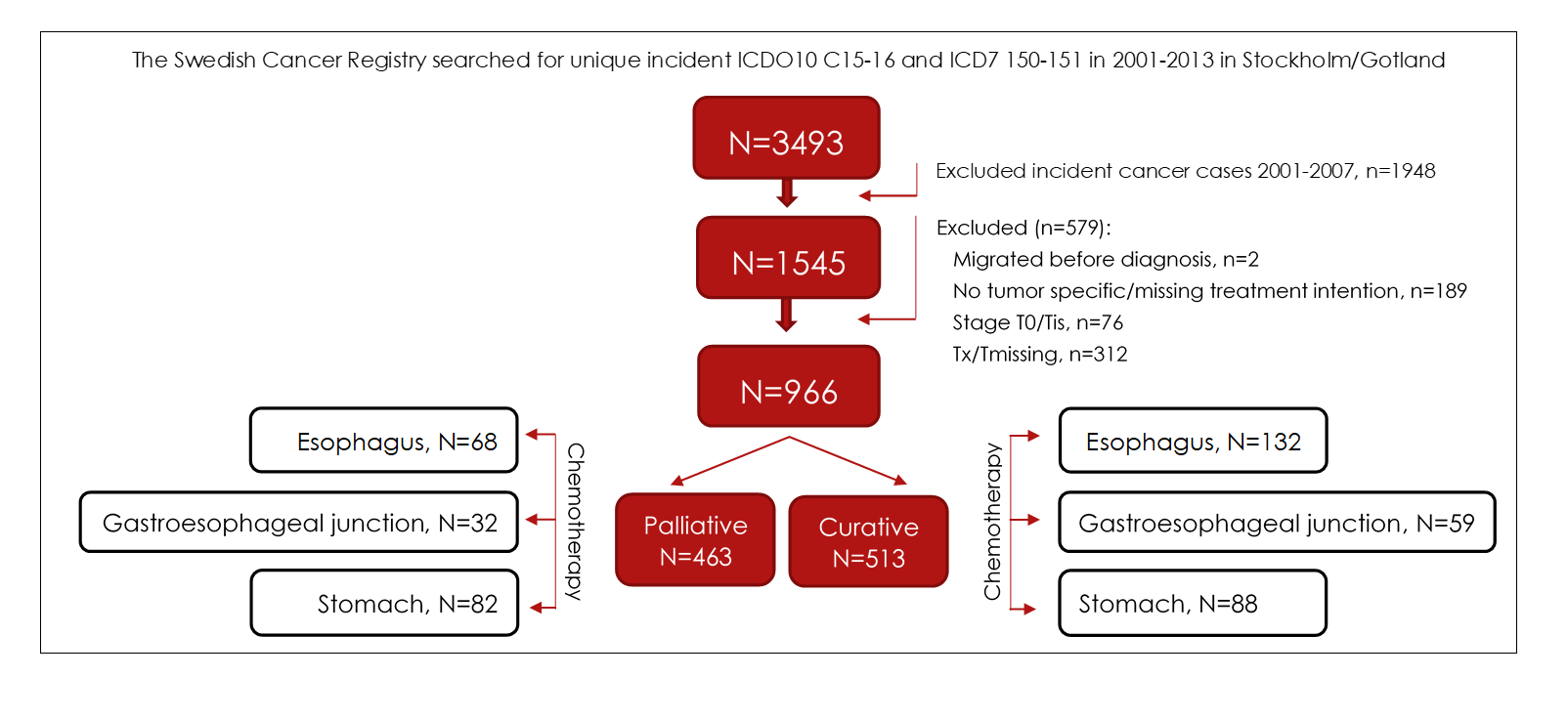

Supplement: Supplementary file 7 — High resolution image (TIF 247 kb). [file 228_2020_2883_MOESM6_ESM.tif]

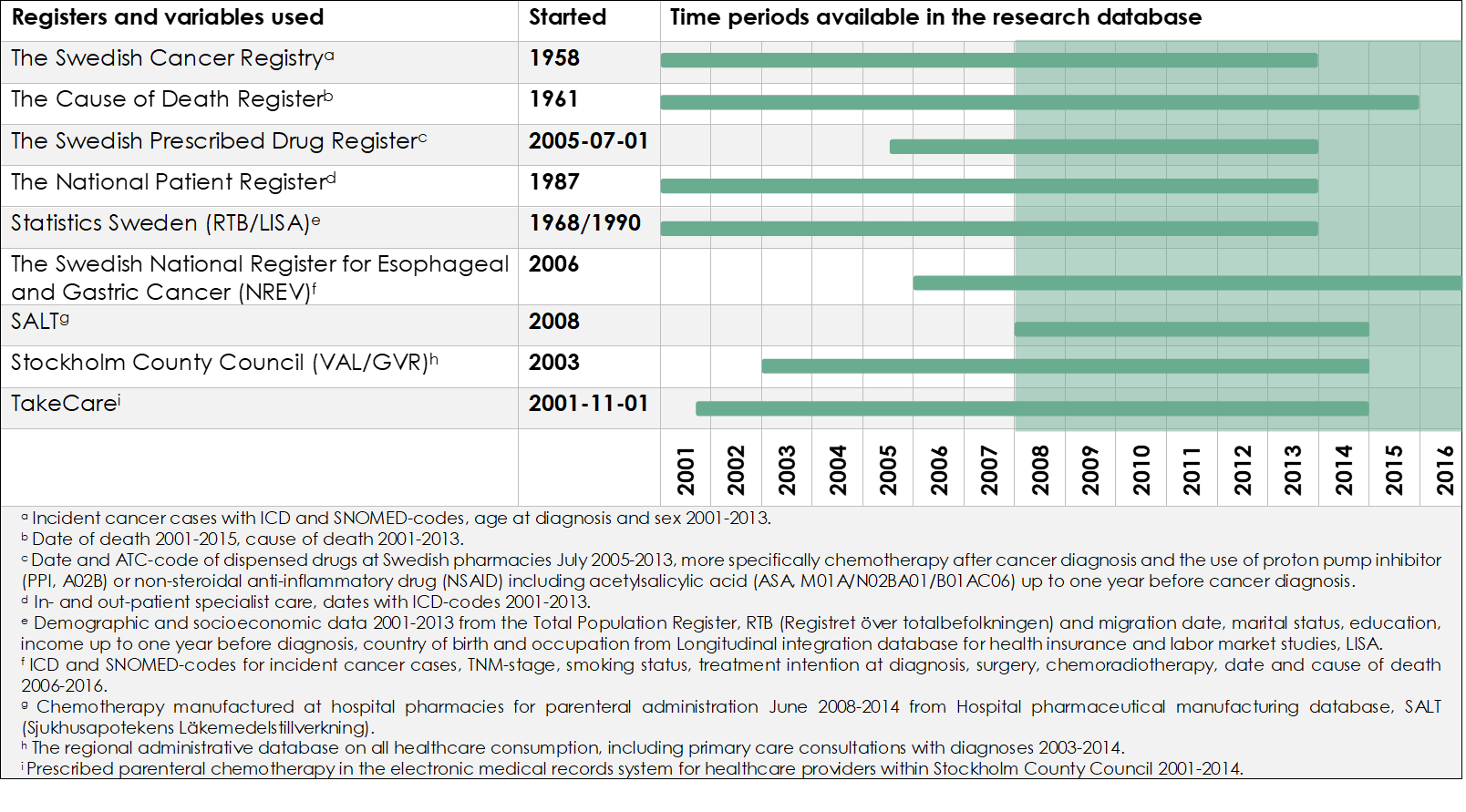

Supplement: Supplementary file 8 — Chart of registries used. Time period used for the construction of the cohort marked with green shade (2008-2016). (PNG 4201 kb). [file 228_2020_2883_Fig6_ESM.png]

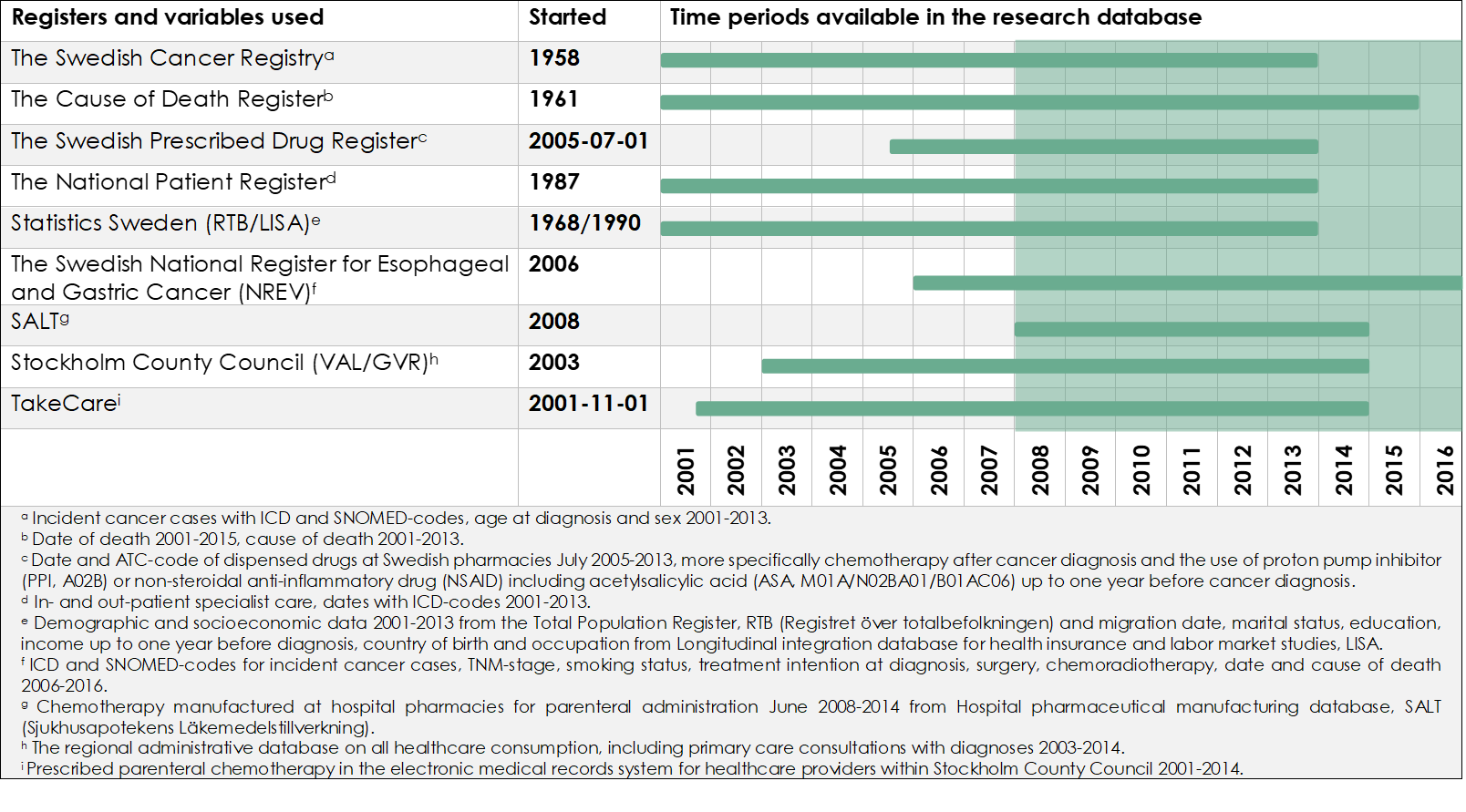

Supplement: Supplementary file 9 — High resolution image (TIF 375 kb). [file 228_2020_2883_MOESM7_ESM.tif]
